# Fluoxetine attenuates the impairment of spatial learning ability and prevents neuron loss in middle-aged APPswe/PSEN1dE9 double transgenic Alzheimer's disease mice

**DOI:** 10.18632/oncotarget.15398

**Published:** 2017-02-16

**Authors:** Jing Ma, Yuan Gao, Lin Jiang, Feng-lei Chao, Wei Huang, Chun-ni Zhou, Wei Tang, Lei Zhang, Chun-xia Huang, Yi Zhang, Yan-min Luo, Qian Xiao, Hua-rong Yu, Rong Jiang, Yong Tang

**Affiliations:** ^1^ Department of Histology and Embryology, Chongqing Medical University, Chongqing, P. R. China; ^2^ Laboratory of Stem Cells and Tissue Engineering, Chongqing Medical University, Chongqing, P. R. China; ^3^ Department of Geriatrics, The First Affiliated Hospital, Chongqing Medical University, Chongqing, P. R. China; ^4^ Department of Physiology, Chongqing Medical University, Chongqing, P. R. China

**Keywords:** fluoxetine, Alzheimer’s disease, cognition, neuron, APP/PS1 mice, Gerotarget

## Abstract

Selective serotonin reuptake inhibitors (SSRIs) have been reported to increase cognitive performance in some clinical studies of Alzheimer’s disease (AD). However, there is a lack of evidence supporting the efficacy of SSRIs as cognition enhancers in AD, and the role of SSRIs as a treatment for AD remains largely unclear. Here, we characterized the impact of fluoxetine (FLX), a well-known SSRI, on neurons in the dentate gyrus (DG) and in CA1 and CA3 of the hippocampus of middle-aged (16 to 17 months old) APPswe/PSEN1dE9 (APP/PS1) transgenic AD model mice. We found that intraperitoneal (i.p.) injection of FLX (10 mg/kg/day) for 5 weeks effectively alleviated the impairment of spatial learning ability in middle-aged APP/PS1 mice as evaluated using the Morris water maze. More importantly, the number of neurons in the hippocampal DG was significantly increased by FLX. Additionally, FLX reduced the deposition of beta amyloid, inhibited GSK-3β activity and increased the level of β-catenin in middle-aged APP/PS1 mice. Collectively, the results of this study indicate that FLX delayed the progression of neuronal loss in the hippocampal DG in middle-aged AD mice, and this effect may underlie the FLX-induced improvement in learning ability. FLX may therefore serve as a promising therapeutic drug for AD.

## INTRODUCTION

Alzheimer's disease (AD) has emerged as the most common cause of dementia in the elderly, characterized by progressive learning dysfunction and memory loss. Some researchers have termed AD as “hippocampal dementia” [1] because the hippocampus is one of the first and most profoundly damaged structures in AD. Amyloid deposits and neurofibrillary tangles, two main pathological hallmarks of AD in the hippocampus, have been found to result in significant hippocampal neuronal dysfunction and death in the clinically detectable stage of AD [2–4]. Moreover, hippocampal neuron loss has been reported to exceed the amyloid deposits and the neurofibrillary tangles in the transgenic mouse model of AD or in patients with AD [5, 6]. Additionally, it has been reported that the decline in cognitive abilities during the late stage of AD appears to more correlate with significant neuron loss than with the amyloid deposits and neurofibrillary tangles in the hippocampus [1, 7, 8]. In fact, many reports have demonstrated that cognitive impairments can be improved by some drugs through the attenuation of neuronal cell death or the rescue of neurogenesis in the hippocampus of AD animal models [9–12]. Still, no effective treatment for AD is currently available. Although several drugs have been approved for the clinical treatment of AD [13], these drugs activate cholinergic or suppress glutamatergic neurotransmission and therefore improve only the symptoms of AD. Their neuroprotective activities remain a topic of debate [14–16].

Fluoxetine (FLX) is a selective serotonin reuptake inhibitor (SSRI) that was approved by the US FDA in 1987 for treating depression. Recently, studies of some clinical types of AD accompanied by depression have demonstrated that FLX enhances cognitive performance in AD patients [17, 18]. However, it is difficult to discern whether the effect of FLX on cognitive improvement in AD patients is due to a direct effect on cognition or is an outcome of mood stabilization. Recently, it has been shown that FLX ameliorates cognitive defects in transgenic AD mice in the early stage of AD [19, 20]. Nevertheless, it remains unknown whether FLX enhances cognitive abilities in transgenic AD mice in the late stage of AD. Moreover, the reason for the protective effect of FLX in AD mice is not completely understood. It is possible that FLX has a protective effect on hippocampal neurons in AD mice. Indeed, it has been demonstrated that FLX not only attenuates deficits in neurogenesis in the hippocampus [21–24], thereby producing a marked improvement in hippocampal-dependent cognitive impairment in neurodegenerative disorders such as Huntington's disease or ischemia [22, 24], but also suppresses hippocampal neuronal death induced by transient global ischemia or certain drugs, resulting in marked improvements in memory impairments in mice or rats [25–27]. We only found one report that addressed the neurogenesis in female mice in a 3xTg model of AD after FLX treatment. In that study, 9-month-old mice in a 3xTg model of AD were given 1 month or 10 months of FLX treatment. At 10 or 19 months, those mice treated with FLX did not show neurogenesis in the DG [28]. However, no study has used unbiased stereological methods to precisely determine the actual total number of neurons in the hippocampus after chronic treatment with FLX in the middle to late stage of AD.

The activation of canonical Wnt signaling pathway develops and maintains the nervous system [29–31]. Recent studies have shown that Wnt signaling is involved in neurodegenerative diseases, especially AD [32–38]. In AD pathology, the level of glycogen synthase kinase-3β (GSK-3β) in AD brains is considerably up-regulated compared to that in healthy brains, while the level of β-catenin is down-regulated [39]. Numerous reports have demonstrated that the activation of Wnt/β-catenin signaling increases hippocampal neurogenesis [40–43] and protects hippocampal neurons from death [44–46] in AD. Furthermore, Li et al. [47] have showed that FLX greatly enhances the phosphorylation of GSK-3β, and Pilar-Cuéllar et al. [48] have revealed that FLX increases the β-catenin level. More recently, Hui et al. [49] have demonstrated that the activation of GSK-3β/β-catenin signaling might be the mechanism by which FLX promotes neurogenesis *in vitro*. Nevertheless, to date, no study has reported whether FLX acts through the canonical Wnt signaling pathway to perform a neuroprotective role in AD. Here, we hypothesized that FLX might play a neuroprotective role in AD *via* the canonical Wnt signaling pathway.

In this study, we evaluated the effect of FLX treatment on APP/PS1 mice in the late stage of AD. The spatial learning and memory abilities of middle-aged APP/PS1 mice were evaluated using a Morris water maze. Subsequently, we accurately determined the number of neurons in the DG and in CA1 and CA3 of the hippocampus in middle-aged AD mice using unbiased stereological techniques. Additionally, we assessed whether the neuroprotective activity of FLX in middle-aged AD mice might be stimulated in part through the activation of canonical Wnt signaling pathway.

## RESULTS

### Fluoxetine alleviates impairment of spatial learning ability in middle-aged APP/PS1 transgenic mice

**Figure 1:**
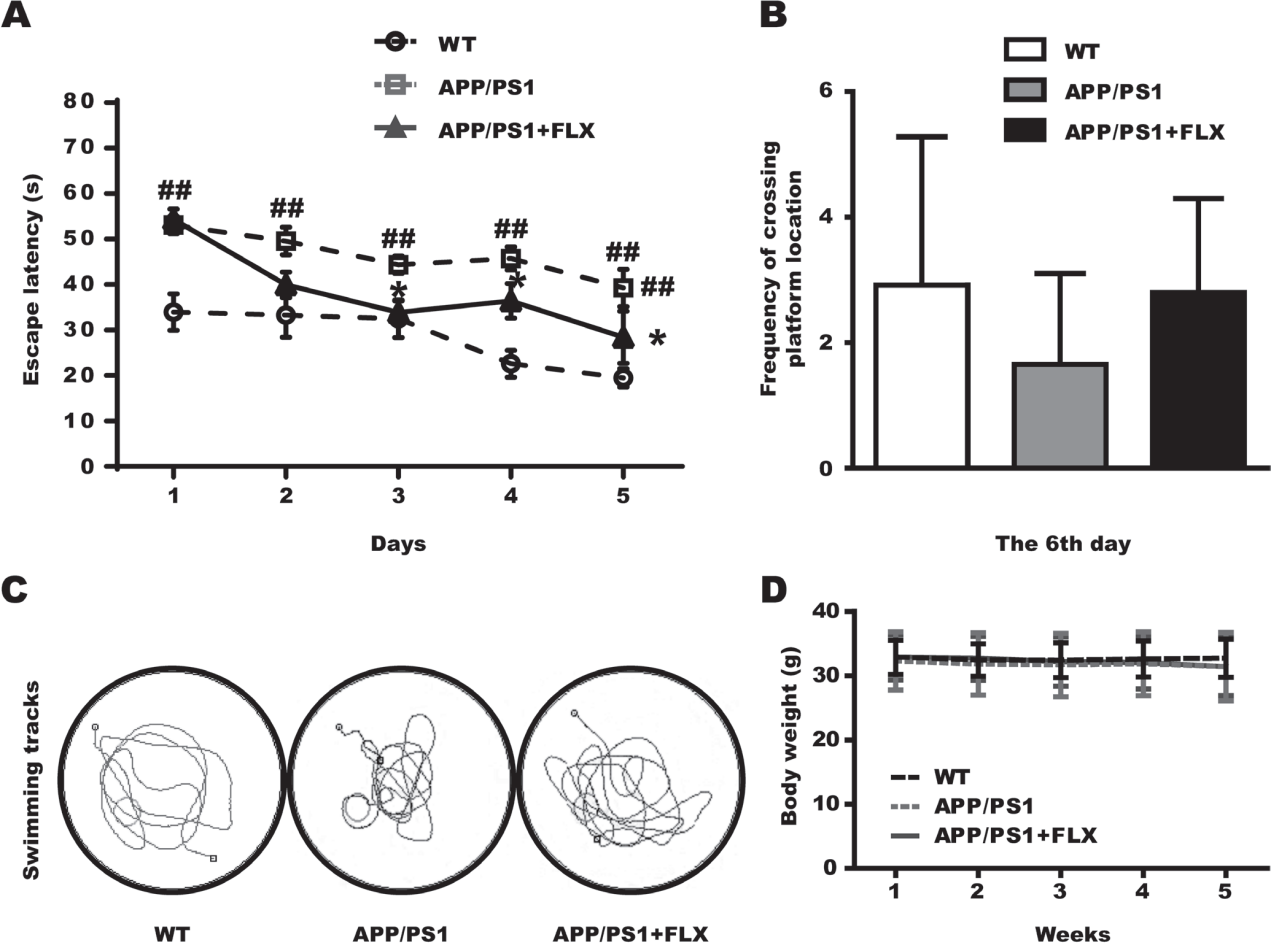
Assessment of the effect of FLX on learning and memory impairment in middle-aged APP/PS1 transgenic mice tested in the Morris water maze and the effect of FLX on body weight of middle-aged APP/PS1 mice FLX was administered (10 mg/kg/day, i.p.) for 4 weeks prior to training in the Morris water maze. Treatment was continued while the mice were submitted to the test. **A**. Mean escape latencies of the three groups (WT, APP/PS1 and APP/PS1+FLX) in the hidden platform tests, which were conducted for 5 consecutive days. **B**. The frequencies at which the mice in the three groups crossed the platform location (on the 6th day) during the probe test. **C**. The swimming tracks the mice in the three groups made in the water tank on the last day of the test. The circle in the left lower quadrant represents the location of the hidden platform, while the curves indicate the different swimming strategies of the three groups of mice. **D**. Body weight of mice in the three groups. The body weight was monitored on a daily basis. Data are presented as the means ± S.E.M. *n* = 10-13/group. ##, *P* < 0.01, *vs*. WT group. *, *P* < 0.05, *vs*. APP/PS1 group.

To investigate whether FLX treatment prevents impairments in learning and memory abilities in AD mice, three groups of mice were subjected to the Morris water maze, which is a well-known test for evaluating hippocampus-dependent learning and memory. In the hidden platform trials, the APP/PS1 mice showed significantly longer escape latencies (Figure 1A, *P* < 0.01). However, the APP/PS1 mice in the FLX-treated (APP/PS1+FLX) group showed significant improvements in learning ability, with escape latencies that were markedly shorter than those of the mice in the APP/PS1 group. (Figure 1A, *P* < 0.05). In the subsequent probe task, the platform was removed, and the mice were then tested to determine whether they were capable of remembering the location of the removed platform. Unexpectedly, the frequency at which the mice crossed the platform location was not statistically different between the APP/PS1+FLX group and their control (APP/PS1) group (Figure 1B, *P* > 0.05), and there was also no significant difference between the WT and APP/PS1 groups (Figure 1B, *P* > 0.05). Despite these results, the FLX-treated APP/PS1 mice were observed to follow tracks that were more similar to those of the WT mice than those of the APP/PS1 controls (Figure 1C). Additionally, the health of the mice was closely supervised, and the body weights of the mice were measured daily during treatment. No significant differences were observed among the three groups of mice (Figure 1D, *P* > 0.05).

### Fluoxetine reduces beta amyloid in the hippocampus in middle-aged APP/PS1 transgenic mice

**Figure 2:**
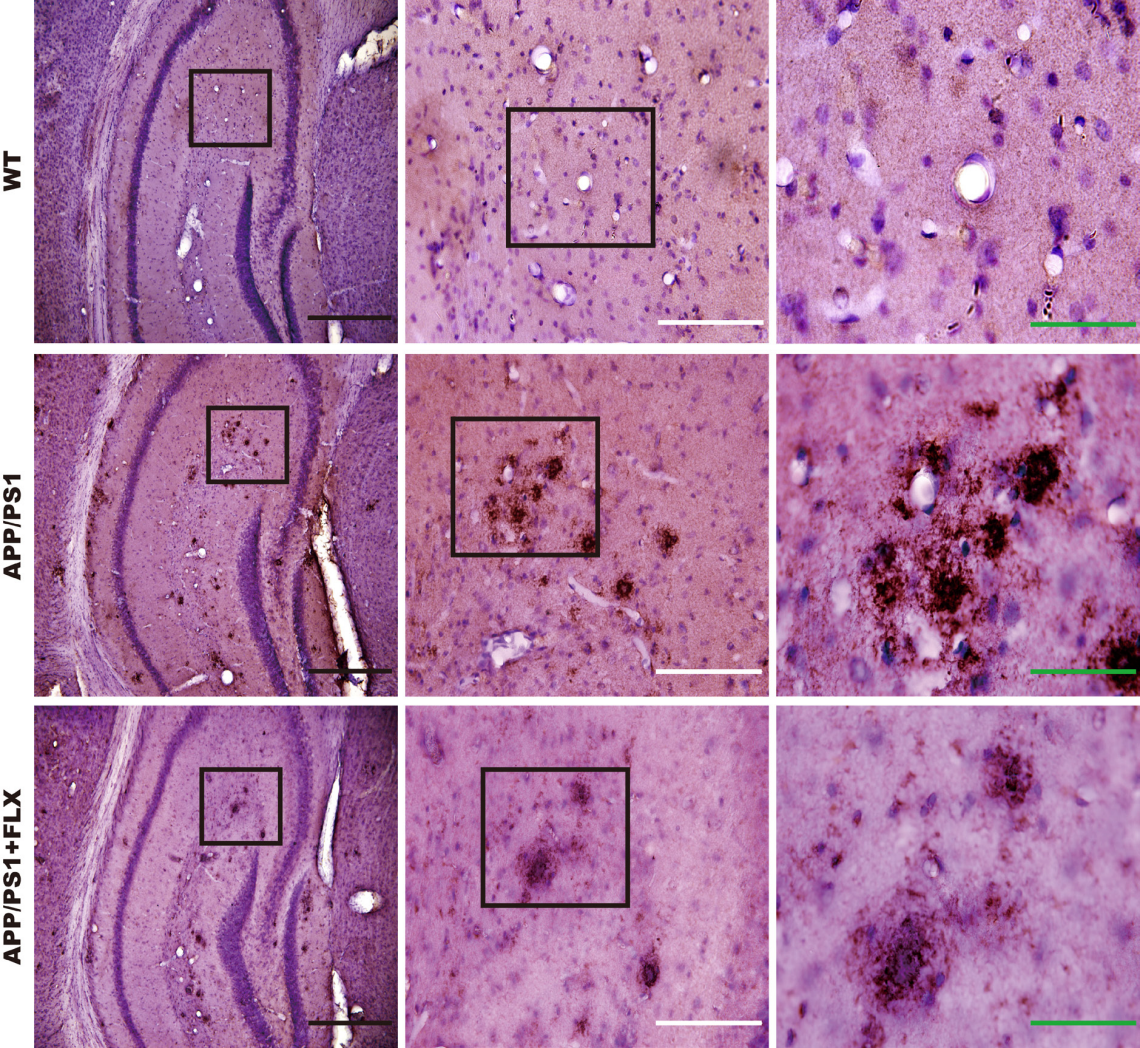
Deposition of beta amyloid in the hippocampus of mice in the WT, APP/PS1 and APP/PS1+FLX groups Immunohistochemical staining for beta amyloid in the hippocampus of mice from the three groups. Enlargements show a higher magnification of the indicated areas of interest. Black scale bars = 500 μm; white scale bars = 200 μm; green scale bars = 80 μm.

One of the major neuropathological hallmarks of AD is the accumulation of beta amyloid in the brain [50]. The brain sections obtained from the APP/PS1 mice that were treated with FLX for 5 weeks were subjected to immunohistochemical analysis to evaluate the deposition of beta amyloid. We found that there was little aggregation of beta amyloid in the hippocampus in the WT mice, but in the APP/PS1 and APP/PS1+FLX mice, beta amyloid was observed to have aggregated in the hippocampus (Figure 2). Moreover, the density of beta amyloid aggregating in the hippocampus was higher and the labeling for beta amyloid was more intense in the APP/PS1 group than in the APP/PS1+FLX group (Figure 2). More specifically, the hippocampal beta amyloid aggregates in the mice in the APP/PS1+FLX group were more randomly scattered and relatively fewer in number.

### Fluoxetine prevents the loss of neurons in the DG but not in the CA1 and CA3 of the hippocampus in middle-aged APP/PS1 transgenic mice

**Figure 3:**
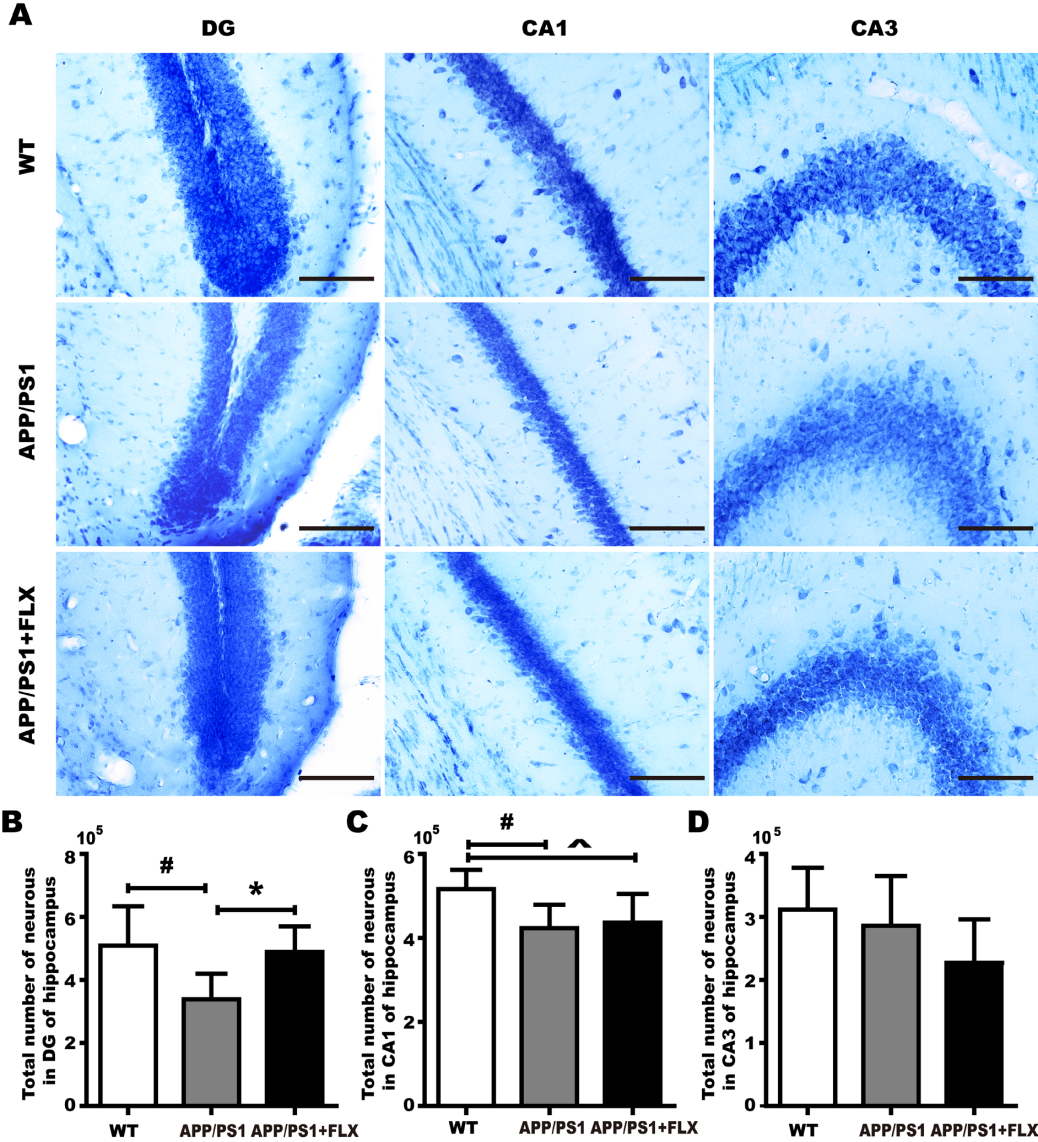
Comparison of accurately determined numbers of neurons in the DG and in CA1 and CA3 of the hippocampus among the WT, APP/PS1 and APP/PS1+FLX groups **A**. representative photographs of toluidine blue-stained tissues were used to show the general distribution of the numbers of neurons in the DG and in CA1 and CA3 of the hippocampus in the WT, APP/PS1 and APP/PS1+FLX groups. **B**. The hippocampal DG in the WT and APP/PS1+FLX mice contained significantly more total neurons than did that in the APP/PS1 mice. **C**. The hippocampal CA1 in the WT mice contained significantly more total neurons than did that in the APP/PS1 and the APP/PS1+FLX mice. **D**. The total numbers of neurons in CA3 of the hippocampus did not significantly differ among the three groups. Data are presented as the mean ± SD. *n* = 5-6/group. #, *P* < 0.05, *vs*. WT group. *, *P* < 0.05, *vs*. APP/PS1 group. ^, *P* < 0.05, *vs*. WT group.

The final cognitive decline in AD is the result of massive cell death [51]. To validate the hypothesis that FLX alleviates cognitive decline in APP/PS1 mice by preventing the loss of neurons in the hippocampus, brain tissues were processed using toluidine blue staining to determine whether FLX increases the numbers of neurons in the DG, CA1 and CA3 of the hippocampus in APP/PS1 mice. We found, using unbiased stereological analysis, that the total number of neurons in the DG of hippocampus in mice in the APP/PS1+FLX group was significantly higher than that in the APP/PS1 group (Figure 3A, 3B, *P* < 0.05). In addition, the total number of neurons in the CA1 of hippocampus in mice in the WT group was significantly higher than that in the APP/PS1 and APP/PS1+FLX group (Figure 3A, 3C, *P* < 0.05). However, the WT, APP/PS1 and APP/PS1+FLX groups showed no significant differences in the total numbers of neurons in the CA3 of hippocampus (Figure 3A, 3D, *P* > 0.05).

### Fluoxetine stabilizes β-catenin and inhibits GSK-3β by inducing the phosphorylation of GSK-3β in the hippocampus of APPswe/PSEN1dE9

**Figure 4:**
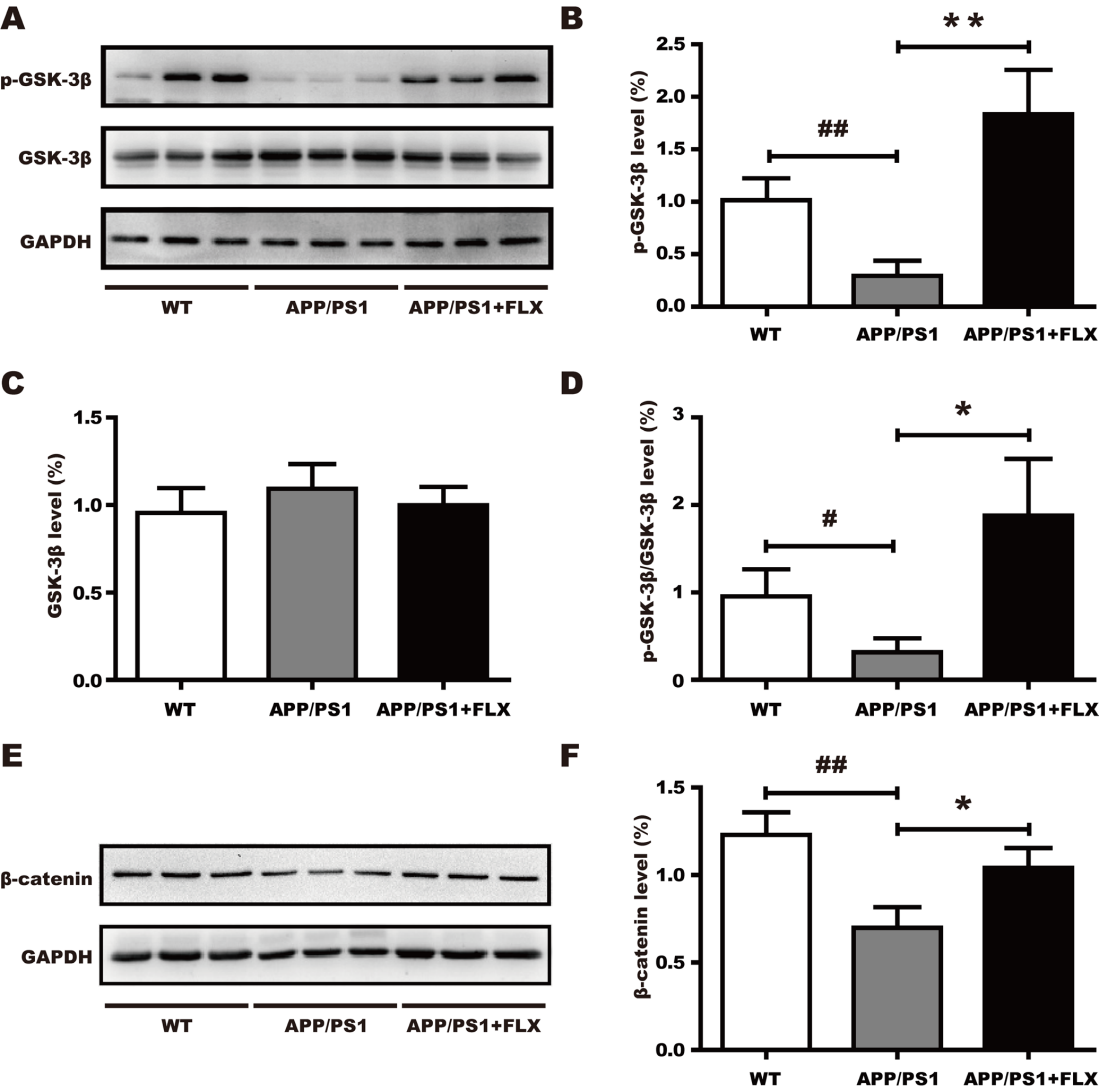
Involvement of the canonical Wnt signaling pathway in the effect of FLX FLX increases the level of phosphorylated of GSK-3β and the level of β-catenin in middle-aged APP/PS1 mice. **A**. Western blots showing the expression levels of p-GSK-3β and total GSK-3β in FLX-treated middle-aged APP/PS1 mice. GAPDH was used as an internal control. **B**.-**D**. Quantification of the levels of GSK-3β and p-GSK-3β, along with the p-GSK-3β/GSK-3β ratio, in middle-aged APP/PS1 mice. **B**. The level of p-GSK-3β was significantly increased in the FLX-treated mice. **C**. There were no significant differences in the levels of total GSK-3β among the three groups. **D**. The p-GSK-3β/GSK-3β ratio was significantly increased in FLX-treated mice. **E**. Western blots showing the expression level of β-catenin in middle-aged APP/PS1 mice after FLX treatments. GAPDH was used as an internal control. **F**. Quantification of the level of β-catenin in middle-aged APP/PS1 mice. The level of total β-catenin was markedly increased after FLX treatment of middle-aged APP/PS1 mice. Data are presented as the mean ± SD. *n* = 5-6/group. ##, *P* < 0.01, *vs*. WT group. #, *P* < 0.05, *vs*. WT group. ** *P* < 0.01, *vs*. APP/PS1 group. *, *P* < 0.05, *vs*. APP/PS1 group.

As recent *in vitro* studies have shown that FLX may activate the canonical Wnt pathway to rescue neuronal loss [49], we investigated whether FLX activates canonical Wnt signaling in APP/PS1 mice. *Via* western blot analysis, the protein levels of GSK-3β, phosphorylated GSK-3β (p-GSK-3β) and β-catenin in the hippocampus were detected in each of the three groups. The level of p-GSK-3β was higher in the APP/PS1+FLX mice than in the APP/PS1 mice (Figure 4A, 4B, *P* < 0.05), but treatment with FLX did not increase the total level of GSK-3β (Figure 4A, 4C, *P* > 0.05). Consequently, treatment with FLX caused the ratio of p-GSK-3β/GSK-3β to be higher than that in the control (Figure 4D, *P* < 0.05). In addition, the level of β-catenin was significantly higher in APP/PS1+FLX mice than in APP/PS1 mice (Figure 4E, 4F, *P* < 0.05). Collectively, these data indicate that treatment with FLX stabilized the level of β-catenin in the hippocampus of APP/PS1 mice.

## DISCUSSION

When AD patients are clinically diagnosed, AD is generally already in a middle to late stage. A growing body of evidence indicates that characteristic pathological changes appear in an APPswe/PSEN1dE9 AD mouse model as early as at 4-6 months of age and increase progressively with age [52–55] and that behavioral deficits are subsequently displayed when the mice are at the age of 10-12 months [56–58]. Therefore, we chose 16-17-month-old APPswe/PSEN1dE9 double transgenic mice [59] to determine whether FLX has a therapeutic effect on the middle-to-late stage of AD, using a paradigm (10 mg/kg/day, i.p., 5 weeks), that has been applied in several previous reports [24, 60–65]. For example, Encinas et al. [61] and Li et al. [24] reported that neurogenesis in the hippocampus was increased by intraperitoneal (i.p.) administration of 10 mg/kg FLX once daily for 15 days or 4 weeks, producing a marked improvement in hippocampal-dependent cognitive impairment. Meanwhile, Malberg et al. [64] demonstrated that chronic (14 or 28 d) but not short-term (1 or 5 d) FLX treatment increased hippocampal neurogenesis. Therefore, we chose the regime of 5 weeks and intraperitoneal (i.p.) administration of 10 mg/kg FLX once daily.

In the present study, evaluation using the Morris water maze showed that treatment with FLX effectively improved the spatial learning ability in cognitively impaired APPswe/PSEN1dE9 transgenic mice that were 16 to 17 months old. Consistent with previous reports, the effect of FLX took time, and the APP/PS1 + FLX mice did not show enhanced spatial learning ability on day 1 [19, 20]. Although FLX had positive effects on the spatial learning ability of APP/PS1 mice, such that animals with FLX remembered the location of the platform better and more quickly than the APP/PS1 mice, animals treated with FLX were still significantly slower than the WT mice, and the spatial learning ability of FLX-treated APP/PS1 mice did not match that of the WT mice. However, our behavioral test failed to detect any significant difference in spatial memory ability between FLX-treated APP/PS1 mice and untreated APP/PS1 mice, in contrast to two previous reports [19, 20]. One previous study showed that when 2-month-old APP_K670N/M671N(Swe)_/PS1_M146L_ AD model mice were treated with FLX at 5 mg/kg/day in their drinking water continuously for 7 months until they reached 9 months of age, these 9-month-old mice displayed significant improvements in spatial learning and memory behaviors in the Morris water maze [20]. Another previous study showed that when 2-month-old APP_K670N/M671N_/PS1_M146L_ AD model mice were treated with FLX at 5 mg/kg/day in their drinking water continuously for 4 months until they reached 6 months of age, and these 6-month-old mice also exhibited significant improvements in spatial learning and memory behaviors in the Morris water maze [19]. The discrepancy in the effect of FLX on the spatial memory ability of AD model mice between our study and these two previous studies may be partly due to the following possible reasons. One reason is the age of our mice; that is, 16- to 17-month-old AD mice may be too old to experience amelioration of their memory abilities. Another reason might be that the dose or the duration of FLX administration may have been too low or slightly too short, such that we may have potentially missed the superior efficacy of FLX by not administering higher doses, not using flexible dosing or not administering the drug for slightly longer. A third possible reason might be that the mice in the different studies expressed different transgenes, which might have arrived at different results.

Because FLX improved the spatial learning in APP/PS1 mice, we were curious as to whether FLX could attenuate the aggregation of hippocampal beta amyloid. Beta amyloid aggregates to form oligomers that are toxic to neurons and are crucial to the progression of AD [66]. The present results showed that FLX reduced the deposition of beta amyloid in the hippocampus of APP/PS1 mice, consistent with previous findings [19, 20, 67, 68], suggesting that the ability of FLX to alleviate cognitive deficits in APP/PS1 mice may depend on decreasing the aggregation of beta amyloid in the hippocampus. However, different researchers have come to conflicting conclusions. Whether reducing beta amyloid is beneficial to the pathogenesis of AD remains controversial, given that several lines of evidence have revealed that drugs targeting to clear the beta amyloid protein plaques fail to improve cognition in AD patients and may even result in terrible side effects, such as skin cancer [69, 70]. Thus, determining whether other potential neurobiological mechanisms may underlie the improvements in cognition observed in the FLX-treated APP/PS1 mice requires further study.

It is widely accepted that spatial learning and memory are closely associated with the hippocampus [71]. The hippocampus is generally considered to consist of the DG, the hippocampus proper, which is composed of the CA1, CA2 and CA3 fields, and the subiculum. Previous studies have suggested that the memory deficits and learning declines observed in elderly AD patients are correlated with neuronal loss in the hippocampus [72, 73]. A growing body of evidence supports the view that attenuation of neuronal loss in the hippocampus improves cognitive impairments in AD models [9, 10]. Therefore, in the current study, we used stereological methods to accurately determine the numbers of neurons in the DG and in CA1 and CA3 of the hippocampus during the late stage of AD to explore whether FLX ameliorates the spatial learning deficit in a middle-aged AD mouse model by protecting neurons in the hippocampus. To our knowledge, this study is the first to adopt stereological methods to accurately assess the number of neurons in the hippocampus in middle-aged APP/PS1 mice that were treated with FLX. Interestingly, we found that the numbers of neurons in the CA1 and DG were significantly lower in the APP/PS1 mice than in the WT mice. The decrease in the numbers of neurons in CA1 and DG is in agreement with previous studies reporting neuronal loss in CA1 [74–78] and DG [1, 77, 78] in AD. However, our data are inconsistent with one recent study [79], which reported that a significant reduction in neurons was only observed in the subiculum of the McGill-R-Thy1-APP transgenic rat model of AD at 18 months of age [79]. This discrepancy may be related to the differences in the transgenes and strains of the animals studied. The McGill-R-Thy1-APP transgenic rats possess only a single transgene, and in these animals, the first Aβ plaque pathology is evident at about 9 months of age in the subiculum [79], while our APPswe/PSEN1dE9 mice show Aβ plaque pathology as early as at 4-6 months of age in the hippocampus [52–55]. Strikingly, Gan et al. [80] have reported that the number of neural progenitor cells is significantly decreased at the stages of Aβ plaque onset and progression. Heggland et al. have considered that it is possible that at older ages, cell loss would become apparent in other brain areas in the McGill-R-Thy1-APP transgenic rat, since the progression of pathology is gradual [79]. They also predicted that although rats, given their larger size as well as more social and complex behaviors, are considered to be physiologically and genetically closer to humans than mice [81], neuronal loss has only been reported in one rat transgenic model, the TgF344-AD line [82], and transgenic mouse models have been more widely used in studies.

Compared with the mice in the APP/PS1 group, the FLX-treated APP/PS1 mice possessed significantly higher numbers of neurons only in the DG. FLX administration had no effect on neurons in the CA1 and CA3 regions of the hippocampus in APP/PS1 mice. Accordingly, we propose that FLX improves the learning ability of APP/PS1 mice by increasing the number of neurons in the DG of the hippocampus. Intriguingly, throughout adulthood, the mammalian brain retains the ability to produce new neurons in two discrete regions, the subgranular zone (SGZ) of the hippocampal DG and the subventricular zone (SVZ) of the lateral ventricle [83–85]. Several studies have shown that adult neurogenesis in the hippocampal DG stimulates hippocampal function and introduces the possibility of a new form of hippocampal plasticity, especially hippocampus-dependent learning and memory [83, 84, 86, 87]. Fitzsimons et al. [88] have reviewed the idea that although the process of adult neurogenesis is up-regulated in the early stage of AD, the neurogenic capacity in AD is clearly insufficient to compensate for the neuronal dysfunction or loss [89, 90]. Furthermore, Demars et al. [91] have reported that APPswe/PS1ΔE9 mice exhibit severe impairments in hippocampal neurogenesis at as early as two months of age. These decreases in neurogenesis within the DG have been associated with cognitive impairments linked with AD [92–95]. In addition, Boekhoorn et al. [96] have reported that proliferation does occur in presenile Alzheimer hippocampus (patients ranging from 63 to 70 years of age), and this proliferation reflects glial and vascular-associated changes in the CA1-3 areas but not neurogenesis in the DG. It is known that 1 month of mouse life is roughly equivalent to 3.54 years of human life; thus, our 16-17-month-old mice is roughly equivalent to 56.64-60.18-year-old humans [97]. Therefore, it is possible that neurogenesis did not occur in the DG of the mice that we used. However, numerous reports have demonstrated that stimulation of adult neurogenesis may be an attractive therapeutic or preventative target to boost the brain's regenerative capacity, and that enhancement of hippocampal neurogenesis might have therapeutic potential in hippocampus-dependent learning and memory in AD [11, 93–95, 98–100]. One recent report has indicated that the improvement of neurogenesis with genetic manipulation can rescue cognitive deficits in 8-9-month-old female APPswe/PSEN1dE9 mice [95]. Our supposition is consistent with this result that FLX might increase the number of DG neurons in APPswe/PSEN1dE9 mice *via* enhancing neurogenesis, thereby improving the spatial learning ability of APPswe/PSEN1dE9 mice. Nevertheless, the increased number of neurons in the DG might be attributed to two main processes: the proliferation of new neurons (neurogenesis) and increased neuronal survival through the attenuation of neuronal cell death [9–12]. The main disadvantage of our current study is that it was difficult to confirm whether FLX increased the number of neurons in the DG by enhancing neurogenesis, by preventing neurons from dying, or through both processes. This question therefore requires further investigation.

It is generally accepted that the inhibition of Wnt signaling is involved in AD neurodegenerative processes [33, 34] and that the activation of Wnt signaling ameliorates neurodegeneration in AD [42, 46]. Importantly, it has been reported that FLX either inhibits GSK-3β activity or enhances β-catenin activity [47, 48]. Furthermore, Valvezan and Klein [101] have reported that p-GSK-3β promotes adult hippocampal neurogenesis, and this might be required for the effects of FLX. Intriguingly, the expression of Wnt-3a, which activates the canonical Wnt signaling pathway, has been reported to be increased by FLX [102]. Interestingly, Pinnock et al. [103] have demonstrated that the neurogenesis-promoting effect of FLX in the rat hippocampus is closely associated with the up-regulation of Wnt-3a. Moreover, Alvarez et al. [104] have reported that the canonical ligand Wnt-3a protects rat hippocampal neurons from death induced by exposure to Aβ. These findings suggest that the neuroprotective effect of FLX might be related to the modulation of canonical Wnt signaling. In the current study, we observed that FLX, a classical SSRI, was involved in the activation of Wnt signaling in middle-aged APP/PS1 AD model mice. The present data revealed that FLX significantly inhibited GSK-3β activity, increased the p-GSK-3β/GSK-3β ratio and stabilized the β-catenin level *in vivo*, indicating that activation of the Wnt signaling pathway might be involved in the neuroprotective effects of FLX that prevent neuronal loss in AD, as has been proposed for the *in vitro* neuroprotective effects [49]. Although these data provide strong evidence of such involvement, it is possible that other pathways, in addition to Wnt signaling, may also mediate the neurogenic effects of FLX *in vivo*. Further study is expected to clarify this issue.

In conclusion, chronic treatment with FLX had a positive effect on cognition in middle-aged APP/PS1 transgenic AD model mice. In addition, this study is the first one to use unbiased stereological methods to accurately investigate the effect of FLX on the numbers of neurons in the DG and in the CA1 and CA3 regions in the middle-aged AD hippocampus. We demonstrated that FLX increased the number of neurons in the hippocampal DG in middle-aged APP/PS1 AD model mice. Furthermore, the mechanism underlying the protective efficacy of FLX is at least partly stimulated by the canonical Wnt signaling pathway. However, additional investigations are required in order to better understand the mechanisms underlying the protective effect of FLX and to determine whether there is an interaction between serotonin and the canonical Wnt signaling pathway. In summary, we demonstrate that FLX might have protective effects on cognition and on neuronal numbers in the hippocampus of middle-aged APPswe/PSEN1dE9 AD mice.

## MATERIALS AND METHODS

### Animals and drug treatment

Male APPswe/PSEN1dE9 double transgenic Alzheimer's Disease mice at the age of 16 to 17 months old and their wild-type (WT) littermates were purchased from the Animal Model Institute of Nanjing University and housed 3-4 per cage, according to *the National Institutes of Health Guidelines for the Care and Use of Laboratory Animals* (NIH Publication No. 85-23). Food and water were made available *ad libitum*, and the mice were housed in a 12 h light-dark cycle at 21-25°C in the Experimental Animal Center of Chongqing Medical University, P. R. China.

**Figure 5:**
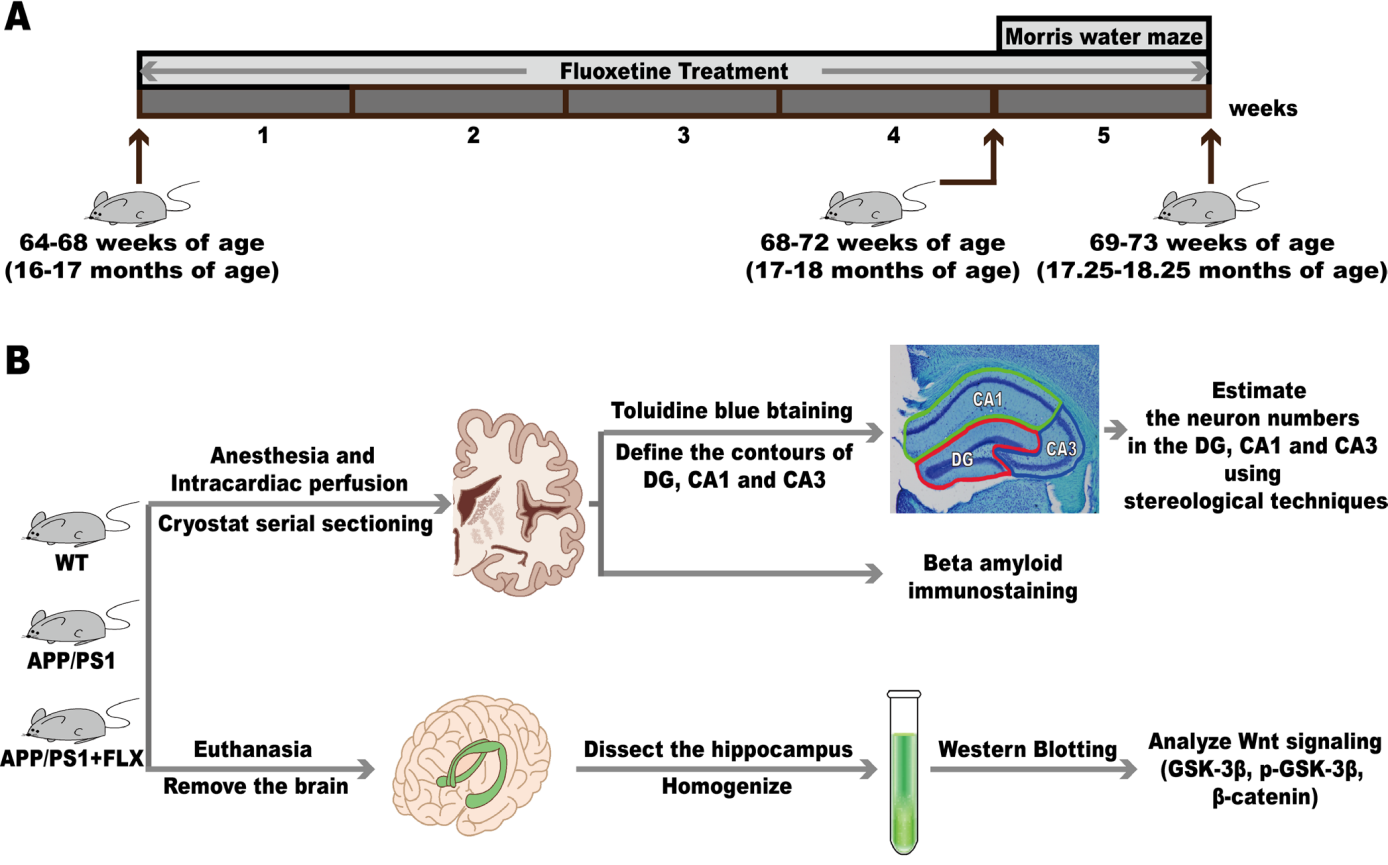
The experimental design **A**. The mice began to receive FLX treatment at 64-68 weeks (16-17 months) of age, and the treatment was continued until they reached 69-73 weeks (17.25-18.25 months) of age, with the FLX administration lasting for 5 weeks. The Morris water maze was conducted when mice were at 68-72 weeks (17-18 months) of age, after being administered fluoxetine for 4 weeks. **B**. Six mice each were randomly chosen from the WT group and the APP/PS1 group, and five mice were randomly chosen from the APP/PS1+FLX group for the subsequent stereological investigation and immunohistochemistry. In each group, those mice not used for the stereological investigation and immunohistochemistry were deeply anesthetized and then killed. Then, the hippocampus was obtained from each brain and homogenized for western blotting analysis.

Forty male APP/PS1 transgenic mice at the age of 16 to 17 months old were randomly assigned to receive systemic administration of either fluoxetine (10 mg/kg i.p. dissolved in 0.9 % NaCl; Sigma, USA) or vehicle (equivalent volume of 0.9 % NaCl i.p.), for 20 mice in the APP/PS1+FLX group and 20 mice in the APP/PS1 group, and 20 male wild-type littermates, the WT group, were assigned to receive systemic administration of vehicle (equivalent volume of 0.9 % NaCl i.p.). The mice began to receive FLX treatment (target dose: 10 mg/kg/day) at 64-68 weeks (16-17 months) of age, and the treatment was continued until they reached 69-73 weeks (17.25-18.25 months) of age, with the FLX administration lasting for 5 weeks (Figure 5A). The regime of 5 weeks and the dose of FLX were chosen based on previous reports [24, 60–65]. All mice were treated once per day in the morning between 9 AM and 10 AM for 5 consecutive weeks.

### Morris water maze

After four weeks of FLX treatment when mice were at 68-72 weeks (17-18 months) of age, during the fifth week of FLX treatment, the spatial learning and memory abilities of the three groups of mice were tested using a Morris water maze, as described previously by our team [105] (Figure 5A). Briefly, the Morris water maze test consists of the hidden platform trials and the probe tasks. Spatial learning ability was tested with the hidden platform trials for five successive days. Each day, a hidden platform trial was initiated by randomly placing a mouse, facing the maze wall, into one of the four quadrants of the maze, with each of the four quadrants used once daily. The trial was terminated automatically when the mouse reached the platform or after 60 s. Then, the mouse was allowed to stay on the platform for 10 s. If the mouse did not find the platform within 60 s, it was guided to the platform. Memory ability was tested the next day, after five days of hidden platform trials, with the probe tasks performed in the same water maze. In the probe tasks, unlike in the hidden platform trials, the platform was removed, and the mice entered the water from two farthest points away from the platform location in two quadrants and were allowed to swim freely for 60 s. In the hidden platform trials, the escape latencies were recorded using a computerized tracking system, while in the probe tasks, the frequency of crossing the platform location and the swimming tracks were monitored. Only 35 mice successfully completed the behavioral experiment (12 mice in the WT group, 13 mice in the APP/PS1 group, and 10 mice in the APP/PS1+FLX group).

### Tissue processing

Stereological investigation and immuno-histochemistry were performed using 6 mice randomly chosen from the WT group and 6 mice randomly chosen from the APP/PS1 group, and 5 mice randomly chosen from APP/PS+FLX group. All 17 of these mice were perfused with cold 4% paraformaldehyde (PFA, pH = 7.4) in phosphate-buffered saline (PBS), and their brains were then removed and fixed in 4% PFA in PBS [106]. After adequate fixation, the left or right hemisphere of each of the total 17 brains was sampled at random. The sampled hemispheres were sectioned frozen on a sliding microtome in the transverse plane at 50 μm, and each brain was collected as 6 separate series in 4% PFA (pH = 7.4). At least one series of brain sections from each mouse was mounted as sectioned and stained with toluidine blue. One or more series of brain sections were immunohistochemically stained for beta amyloid using a mouse monoclonal antibody (Abcam, Cambridge, England). (Figure 5B)

In each group, the mice not used for the stereological investigation and immunohistochemistry were deeply anesthetized *via* an i.p. injection of 1% pentobarbital sodium and then sacrificed by cervical dislocation, and their brains were promptly removed. The hippocampus was obtained from each brain and homogenized *via* sonication on ice in lysis buffer. Then, the homogenate was centrifuged at 12000 g for 15 min at 4 °C. The supernatants were collected, and the total protein levels were measured by using a BCA protein assay kit. Then, the supernatants were heated to 95-100 °C for 5 min, cooled on ice for a little while, and then stored at 4 °C until western blot analysis. (Figure 5B)

### Immunohistochemistry

Sections were rinsed 3 times in 0.01 M PBS/0.3% Triton X-100 and then incubated in 3% H_2_O_2_ for 15 min with gentle agitation. The sections were then blocked to prevent nonspecific antigen binding in 4% BSA/0.1% Triton X-100/PBS for 1 h at room temperature. Blocking was followed by overnight incubation with primary antibodies (anti-amyloid, 1:50; or anti-actin, 1:1000; see Table 1) at 4 °C. The sections were then rinsed 3 times in 0.1 M PBS and incubated with biotinylated horse anti-mouse IgG (1:500 in PBS, ZSGB-BIO, SP-9002) for 1 h at room temperature. Subsequently, the sections were treated with streptavidin avidin-biotin enzyme complex (S-A/HRP, ZSGB-BIO, SP-9002) for 1 h. The labeling was visualized using 3,3-diaminobenzidine (DAB, ZSGB-BIO, ZLI-9018). The sections were rinsed, successively dehydrated in 95% and 100% ethyl alcohol, cleared in xylene and coverslipped with Permount in a fume hood. In addition, some of the sections were stained using the same procedure without beta amyloid antibody to verify the staining specificity of the antibody.

**Table 1:** Detailed information on the antibodies used in this study

Antibody	Isotype	Type	Dilution	Source
Beta Amyloid	Mouse IgG	Mono-	1: 50 for IHC	Abcam (ab11132)
GSK-3β	Rabbit IgG	Mono-	1:1000 for WB	CST (#12456)
pS9-GSK-3β	Rabbit IgG	Mono-	1:1000 for WB	CST (#9323)
β-Catenin	Rabbit IgG	Mono-	1:1000 for WB	CST (#9323)
GAPDH	Rabbit IgG	Mono-	1:1000 for WB	CST (#5174)

Mono-, monoclonal; IHC, immunohistochemistry; WB, Western-blotting; p, phosphorylated; CST, Cell Signaling Technology.

### Stereology

#### General

Unbiased stereological estimates of neuron numbers were obtained by using a stereology system (New CAST, Denmark). An Olympus Optiphot microscope was equipped with a motorized stage that allowed precise, automatic movements in the X and Y directions (ProScan, H101A, Britain), a video camera (Olympus, DP71, Japan) to project images onto the computer screen, and a microcator (ProScan, Britain) that was attached to the stage to obtain a precise measurement of the focal depth (in 0.1 μm). The total numbers of neurons in the DG and in CA1 and CA3 of the hippocampus were estimated under a 100x oil objective lens (N.A. 1.40) using the optical fractionators [107].

#### Anatomical boundaries of the DG, CA1 and CA3

Stereological analysis was performed using six mice randomly chosen from the WT group, six mice randomly chosen from the APP/PS1 group, and five mice randomly chosen from the APP/PS1+FLX group. The left or right hemisphere was chosen randomly from each mouse. Each hemisphere was cut along the coronal plane, from the caudal cut surface of every brain slice, into 50-μm-thick successive slabs (12 ± 0.5 slabs/hemisphere), followed by staining with toluidine blue. Each whole slab was scanned under low magnification (4x objective). While using the 4x objective lens, contours were traced around the edges of the DG, CA1 and CA3, based on the borders shown in Paxinos and Franklin's mouse brain atlas [108] and a report by Heggland et al. [79]. The borders were determined using cytoarchitectonic features in sections stained with toluidine blue.

**Figure 6:**
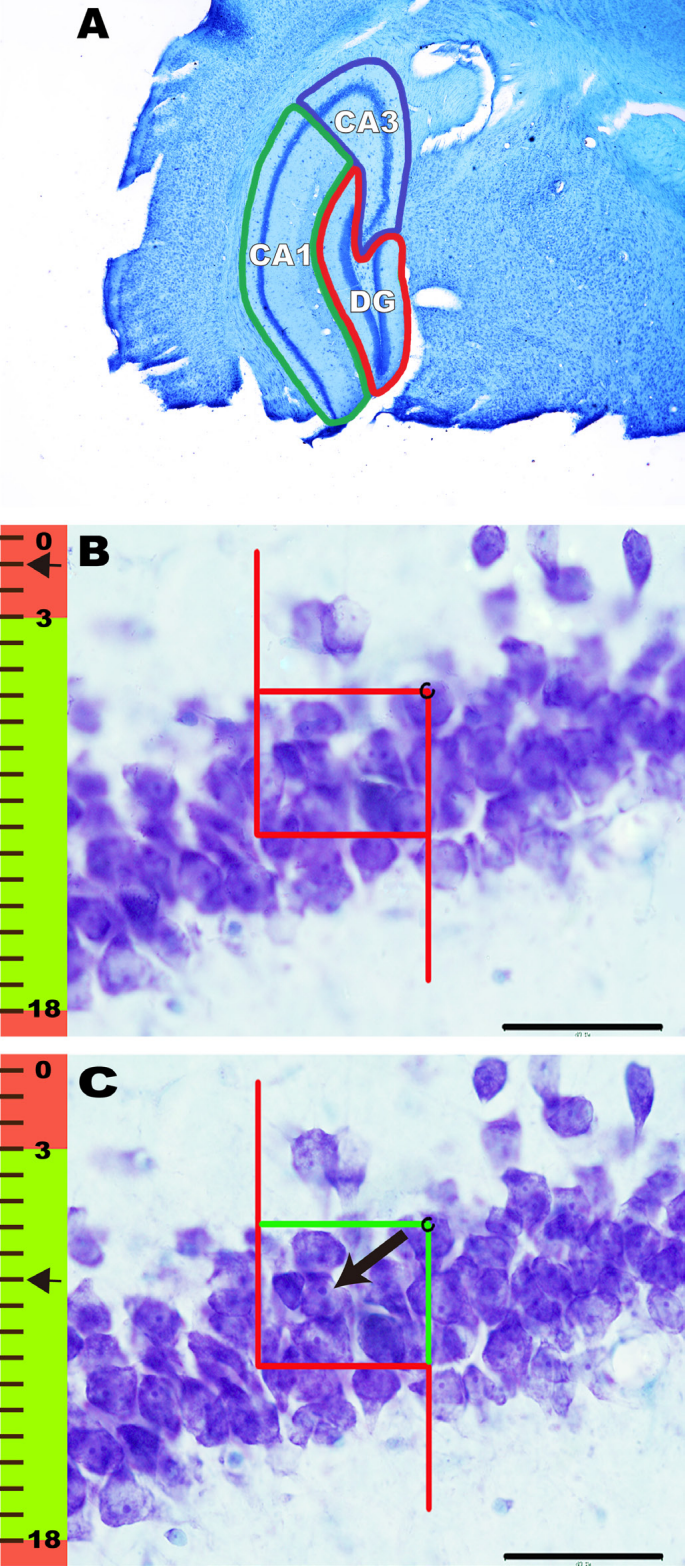
The contours of the DG, CA1, and CA3 of the hippocampus and the method of counting neurons **A**. Coronal histological section showing the contours of the DG (red), CA1 (green), and CA3 (blue) in the hippocampus. **B**.-**C**. The method of counting neurons. The focal depth in micrometers (measured using a microcator attached to the microscope stage) is indicated on the focal depth bar, where 0 m represents the top surface of the section. An unbiased counting frame is included in **B**. and **C**. The red line of the frame and its extension represent the exclusion lines, and the green line of the frame represents the inclusion line. Neurons were counted when their nuclei first came into focus if they were completely inside of the counting frame or partly inside the counting frame but only touching the inclusion (green) line. **B**. When the microscopic field of vision was clearly focused for the first time, its location on the Z axis was set to 0 μm. Then, as the microscope was adjusted, the neurons within the guard zones were not counted. **C**. Thereafter, as the Z axis moved below the guard zones, focusing downward to 8.0 μm brought 1 nucleolus into focus, as indicated by the arrow.

The DG region, which is horseshoe-shaped in cross-section, contains the most densely packed and smallest neuronal cell bodies in the hippocampus. Because the layers in the DG are not in immediate contact with layers in other regions, the DG can be easily defined (Figure 6A). The CA1 region can be roughly defined from CA2 subfield and the prosubiculum. The pyramidal neurons in CA1 are thin, homogeneously sized and smaller than those in the CA2 and CA3 regions (Figure 6A). The CA3 region is divided into two main components that are roughly the same in size. The regio inferior is a tightly packed layer. The end of the regio superior is defined by a transition zone containing large pyramidal neurons that are similar to those in the regio inferior but more loosely arranged (Figure 6A) [79, 107].

### Estimation of neuronal numbers in the DG and in CA1 and CA3

Under an oil objective lens, an unbiased counting frame was superimposed on each optical section. The area of the unbiased counting frame was set as 913.65 μm^2^ for neurons in the DG and CA1 and CA3 regions of the hippocampus. Since every six sections were sampled in a systematic and random fashion, the section sampling fraction (ssf) was 1/6. The area sampling fraction (asf) was set to 0.0004. The dissector height was set to 15 μm and was centered in the tissue to ensure that the heights of guard zones on the top and bottom surfaces of the tissue were 3 μm. The average section thickness was 19 μm. Therefore, the fraction of the section thickness (tsf) was equal to 15/19.

Based on the above parameters set in the stereology analysis system, a three-dimensional probe (optical dissector) was used to directly count the number of neuronal nuclei in a known fraction of the volume of the DG, CA1 or CA3 (Figure 6A). When the microscopic field of vision was clearly focused for the first time, its location on the Z axis was set to 0. Then, as the microscope was adjusted, the neurons within the guard zones were not counted (Figure 6B). Thereafter, as the Z axis moved below the guard zones, if the nuclei of neurons came into focus and did not touch the forbidden lines of the unbiased counting frame, those nuclei were counted (Figure 6C). Glial cells were not counted, and they were differentiated from neurons based on morphological criteria, especially the absence of a large nucleus. The precise rules for sampling and obtaining unbiased estimates of the number of neurons, N, have been described previously [79, 107, 109, 110].

N(total) = ΣQ^—^ × 1/ssf × 1/asf × 1/tsf

where N is the total number of neurons in the hippocampal DG, CA1 or CA3. ΣQ^—^ denotes the total number of neurons counted in the DG, CA1 or CA3 of the hippocampus per mouse.

### Western blot analysis

The hippocampus obtained from the mice were homogenized using sonication on ice in a lysis buffer containing 200 mM PMSF in DMSO, a protease inhibitor cocktail in DMSO, 100 mM sodium orthovanadate in water, and phosphatase inhibitor cocktails. Then, the homogenates were centrifuged at 12000 g for 15 min at 4°C, and the protein concentrations of the supernatants were then quantified by using BCA assays.

Protein samples (45 μg or 80 μg/sample) were electrophoresed using 8%, 10% or 12% SDS-PAGE and then transferred onto PVDF membranes (Millipore, USA). Next, the membranes were blocked in 5% bull serum albumin (BSA) at 37°C for 2 h and then incubated in primary antibodies against GSK-3β, p-GSK-3β or β-catenin at 4°C overnight (see Table 1). On the following day, after being thoroughly washed with TBST (TBS with 0.1% Tween 20), each membrane was incubated with the respective HRP-conjugated secondary antibody at 37°C for 1 h. The protein bands were visualized by using an ECL Prime kit (Millipore, USA).

### Statistical analysis

The data are presented as the mean ± standard deviation (SD) unless otherwise noted. The SPSS statistical software package (ver. 22.0, SPSS Inc., Chicago, USA) or Prism statistical software version 6.0 (GraphPad Software, La Jolla, CA, USA) was used to compare differences among groups *via* repeated-measures analysis of variance (ANOVA) or one-way ANOVA followed by either the LSD post hoc test or the Games-Howell multiple comparisons post hoc test. A value of *P* < 0.05 was accepted as statistically significant. The coefficient of error (CE) estimates were determined according to Gundersen et al [111].
